# Mechanistic Insights Into the Association Between Gut Microbiota Diversity and Atherosclerosis, Acute Coronary Syndrome, and Peripheral Arterial Disease Progression

**DOI:** 10.1002/clc.70455

**Published:** 2026-08-26

**Authors:** Amr Ali Mohamed Abdelgawwad El‐Sehrawy, Usamah Sayed, Zarina Nasirova, Quvonch Tursunov, Omer Qutaiba B. Allela, Lara Asim Kzar, Rajashree Panigrahi, Neeraj Bainsal

**Affiliations:** ^1^ Internal Medicine, Diabetes, Endocrinology and Metabolism Mansoura University Mansoura Egypt; ^2^ Faculty of Allied Medical Sciences, Hourani Center for Applied Scientific Research Al‐Ahliyya Amman University Amman Jordan; ^3^ Department of Internal Diseases and Cardiology No. 2 Samarkand State Medical University Samarkand Uzbekistan; ^4^ Department of Basic Medical Sciences Termez University of Economics and Service Termez Uzbekistan; ^5^ College of Pharmacy Alnoor University Mosul Iraq; ^6^ Department of Pharmacy College of Pharmacy, The Islamic University Najaf Iraq; ^7^ Department of Microbiology, IMS and SUM Hospital Siksha ‘O’ Anusandhan Bhubaneswar Odisha India; ^8^ University Institute of Pharma Sciences Chandigarh University Mohali Punjab India

**Keywords:** acute coronary syndrome, atherosclerosis, cardiovascular disease, dysbiosis, gut microbiome, lipopolysaccharide, peripheral arterial disease, SCFA

## Abstract

**Background:**

The gut microbiome has emerged as a potential contributor to cardiovascular diseases (CVDs), including atherosclerosis, acute coronary syndrome (ACS), and peripheral arterial disease (PAD). While observational studies link dysbiosis to CVD, causal relationships remain uncertain.

**Methods:**

This narrative review synthesizes evidence from human observational studies, clinical interventions, and experimental models to distinguish association from mechanistic plausibility and clinical causality. Literature was searched through July 2026 in PubMed/MEDLINE, Web of Science, and Scopus.

**Results:**

Microbial metabolites—including trimethylamine N‐oxide (TMAO), short‐chain fatty acids (SCFAs), bile acids, and lipopolysaccharide (LPS)—modulate endothelial function, immune cell programming, platelet activity, and plaque stability through receptor‐mediated signaling and epigenetic regulation. SCFAs demonstrate potentially protective effects via GPCR and HDAC pathways, while TMAO is associated with atherothrombotic risk. However, much mechanistic evidence derives from preclinical studies. Heterogeneity from diet, geography, host characteristics, renal function, and medications substantially influences microbiota‐CVD associations.

**Conclusion:**

The gut‐vascular connection is biologically plausible, but definitive clinical causality remains unproven. Microbiome‐directed therapies (dietary modulation, pre/pro/synbiotics, targeted metabolite inhibition) are investigational. Prospective, standardized, adequately powered human studies with clinically meaningful outcomes are essential before routine cardiovascular application.

## Summary

- Observational studies associate gut dysbiosis and altered microbial diversity with atherosclerotic disease phenotypes, but these signatures vary across cohorts and do not establish causality.
- Barrier disruption, LPS translocation, and pro‐inflammatory immune signaling provide plausible links between dysbiosis and vascular injury, with much of the mechanistic evidence derived from experimental and translational studies.
- SCFAs have potentially protective effects through GPCR and HDAC pathways, whereas higher TMAO is associated with atherothrombotic risk and has experimentally demonstrated effects on endothelial and platelet biology.
- Diet, geography, age, sex, renal function, comorbidities, and concomitant medications can substantially modify gut microbial composition and confound microbiome‐CVD associations.
- Microbiome‐directed therapies remain investigational; adequately powered clinical outcome trials are needed before routine cardiovascular use.

## Graphical abstract

Proposed gut‐vascular framework linking microbial ecology to atherosclerotic disease. Diet and related exposures shape dysbiosis or microbial resilience; resulting changes in SCFAs, TMAO, bile acids, and LPS may modulate endothelial function, immune activation, and platelet activity, contributing to atherosclerosis, ACS, and PAD. Arrows indicate proposed or experimentally supported pathways and should not be interpreted as proof of clinical causality.

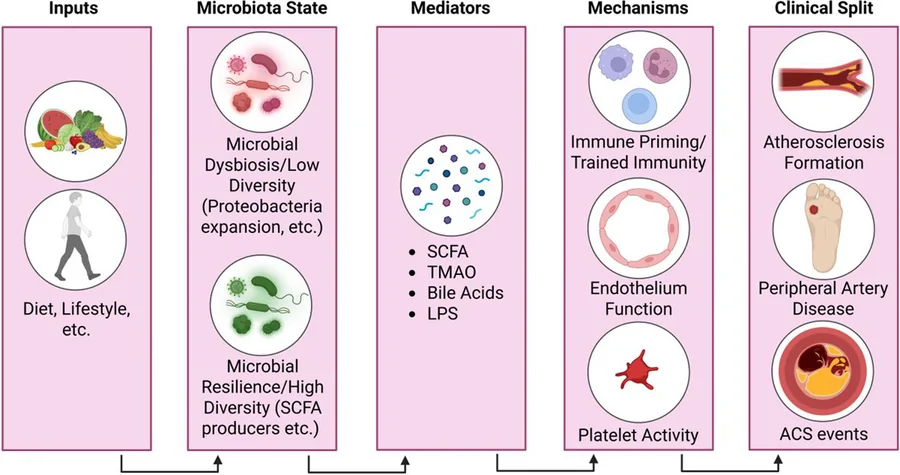

**Abbreviations**
ACS: acute coronary syndrome
AHR: aryl hydrocarbon receptor
CAD: coronary artery disease
CRP: C‐reactive protein
CVDs: cardiovascular diseases
DNMTs: DNA methyltransferases
ECs: endothelial cells
eNOS: endothelial nitric oxide synthase
FMT: fecal microbiota transplantation
FXR: Farnesoid X receptor
GALT: gut‐associated lymphoid tissue
GPCRs: G‐protein coupled receptors
HDACs: histone deacetylases
hsCRP: high‐sensitivity CRP
HSPC: hematopoietic stem/progenitor cell
ICAM‐1: intercellular adhesion molecule‐1
ImP: imidazole propionate
IPA: indole‐3‐propionic acid
IRS1: insulin receptor substrate
LBP: LPS‐binding protein
LDLs: low‐density lipoproteins
LPS: lipopolysaccharides
MACE: major adverse cardiac events
miRNA: microRNA
mRNAs: messenger RNAs
NF‐κB: nuclear factor kappa‐light‐chain‐enhancer of activated B cells
NO: nitric oxide
NSTEMI: non‐ST‐segment elevation myocardial infarction
PAD: peripheral arterial disease
PAG: phenylacetylglutamine
PAMPs: pathogen‐associated molecular patterns
PPT: personalized postprandial‐targeting
PVAT: perivascular adipose tissue
SAM: S‐adenosylmethionine
SCFAs: short‐chain fatty acids
SMCs: smooth muscle cells
STEMI: ST‐segment elevation myocardial infarction
TET: ten‐eleven translocation
TF: tissue factor
TLRs: toll‐like receptors
TMA: trimethylamine
TMAO: trimethylamine N‐oxide
Tregs: regulatory T cells
VCAM‐1: vascular cell adhesion molecule‐1
VSMC: vascular smooth muscle cell
vWF: Von Willebrand factor

## 1 Introduction

Cardiovascular diseases (CVDs), encompassing atherosclerosis, acute coronary syndrome (ACS), and peripheral arterial disease (PAD), constitute a formidable global health challenge, representing a primary cause of morbidity and mortality worldwide. While traditional risk factors such as hypertension, hyperlipidemia, and diabetes are well‐established, a growing body of evidence implicates the gut microbiota as a significant, yet complex, contributor to the pathogenesis and progression of these conditions [1]. The intricate interplay between the host and its microbial inhabitants, particularly within the gastrointestinal tract, extends beyond local digestion, influencing systemic metabolic and immune processes that directly affect cardiovascular health [2].

The human gastrointestinal tract harbors a complex and dynamic ecosystem of microorganisms, collectively termed the gut microbiota. This community, predominantly bacterial, comprises over 100 trillion microbial cells and exhibits genomic variation orders of magnitude greater than the human genome [3]. Microbial diversity, often assessed by alpha‐diversity metrics (e.g., Shannon index, Chao1 index) and beta‐diversity, reflects the richness and evenness of species within an individual and the compositional differences between individuals, respectively. These microbial inhabitants engage in a symbiotic relationship with the host, contributing significantly to numerous physiological processes [4]. These contributions include aiding in nutrient digestion, synthesizing vitamins, protecting against pathogen colonization, and modulating immune system functions [5]. A balanced and diverse gut microbiota is generally associated with robust health, whereas dysbiosis, an alteration in composition and metabolic function, is increasingly linked to various pathologies, including CVDs [6]. While some studies indicate no significant difference in overall microbiota diversity among healthy controls and patients with high blood pressure or coronary heart disease, variations in richness and the dominance of specific bacterial taxa are frequently observed [7]. For example, individuals with coronary heart disease may exhibit a dominance of Bacteroidetes and Bacteroidia, distinct from the profiles seen in healthy populations or those with hypertension. These compositional shifts often correspond with changes in metabolic output, influencing host physiology [8].

Atherosclerosis is a chronic, progressive inflammatory disease characterized by the accumulation of lipids, fibrous tissue, and immune cells within the arterial walls, leading to plaque formation. This process, initiated by endothelial dysfunction, involves complex interactions between oxidized low‐density lipoproteins (LDLs), macrophages, T cells, and smooth muscle cells [9]. Over time, atherosclerotic plaques can grow, narrow arteries, and restrict blood flow, forming the basis for many cardiovascular events. ACS represents a spectrum of conditions, including unstable angina, non‐ST‐segment elevation myocardial infarction (NSTEMI), and ST‐segment elevation myocardial infarction (STEMI) [10]. ACS typically results from the rupture or erosion of an atherosclerotic plaque, leading to thrombus formation and partial or complete occlusion of a coronary artery. This acute reduction in blood supply to the myocardium causes myocardial ischemia and potentially necrosis. The severity and clinical manifestation depend on the extent and duration of ischemia [11]. PAD involves atherosclerosis affecting arteries outside of the heart and brain, most commonly those supplying the limbs. Similar to coronary artery disease, PAD results from plaque accumulation, leading to arterial narrowing and reduced blood flow to the extremities, particularly the legs [12]. Symptoms range from intermittent claudication (pain during exercise) to critical limb ischemia, which can result in tissue loss and amputation. The underlying pathological processes in PAD mirror those in coronary atherosclerosis, driven by inflammation, lipid dysregulation, and endothelial injury. Common to all these conditions are shared risk factors such as dyslipidemia, hypertension, diabetes, smoking, and chronic inflammation [13]. An understanding of the contribution of gut microbiota to these interconnected pathologies is gaining traction, suggesting that microbial influences may represent a modifiable factor in disease prevention and management.

Accumulating evidence suggests that the gut microbiota may influence the onset and progression of CVDs through various pathways. The gut microbiota acts as a metabolic organ, producing bioactive metabolites that interact with host physiology, affecting cardiovascular homeostasis [14]. Dysbiosis, characterized by alterations in microbial composition, can contribute to pathologies such as atherosclerosis, hypertension, and heart failure [15]. Gut microbes metabolize dietary compounds to generate various metabolites, including trimethylamine N‐oxide (TMAO), short‐chain fatty acids (SCFAs), bile acids, and phenylacetylglutamine (PAG) [16]. These metabolites can exert either pro‐atherogenic or cardioprotective effects. The gut microbiota profoundly influences the host immune system, including gut‐associated lymphoid tissue (GALT) and systemic inflammatory responses [17]. Dysbiosis can promote chronic low‐grade inflammation, a hallmark of atherosclerosis. Impaired intestinal barrier function, often associated with dysbiosis, can lead to increased translocation of bacterial products, such as lipopolysaccharides (LPS), into the systemic circulation. This endotoxemia can heighten inflammation and contribute to cardiovascular damage [18].

This review synthesizes current evidence on the association between gut microbiota diversity and the progression of atherosclerosis, ACS, and PAD, while distinguishing observational associations from mechanistic and interventional evidence. The focus is on cellular, molecular, and epigenetic pathways that may explain observed clinical relationships without assuming that every association is causal. By integrating these evidence levels, the review aims to identify biologically plausible pathways, areas of uncertainty, and clinically relevant knowledge gaps that can guide future mechanistic and interventional research.

## 2 Methodological Considerations

This article is a narrative mechanistic review rather than a systematic review or meta‐analysis. For the present revision, the literature search was updated through July 31, 2026. Peer‐reviewed publications were sought in PubMed/MEDLINE, Web of Science, and Scopus, with older seminal studies retained when they were necessary to establish a foundational mechanism or therapeutic concept. The review focused on atherosclerosis, ACS, and PAD, while related cardiometabolic studies were considered when they directly informed a mechanism relevant to these vascular phenotypes.

Search concepts were combined using Boolean operators and included: (“gut microbiota” OR microbiome OR dysbiosis OR diversity) AND (atherosclerosis OR coronary artery disease OR acute coronary syndrome OR myocardial infarction OR peripheral arterial disease OR cardiovascular disease) AND (mechanism OR metabolite OR inflammation OR epigenetic OR TMAO OR short‐chain fatty acid OR bile acid OR LPS OR probiotic OR prebiotic). Targeted secondary searches were performed for specific pathways and interventions, including endothelial dysfunction, trained immunity, platelet activation, microbial enzyme inhibition, fecal microbiota transplantation, phage or live‐biotherapeutic approaches, and registered cardiovascular microbiome trials. ClinicalTrials.gov was additionally checked for ongoing or planned studies relevant to the translational section.

Eligible evidence included peer‐reviewed human observational studies, prospective cohorts, clinical interventions or randomized trials, systematic reviews with direct relevance to the review question, and well‐controlled animal or cellular studies that provided mechanistic information not obtainable in humans. Studies were excluded when they addressed unrelated cardiovascular conditions without a relevant gut‐vascular mechanism, reported only a microbial association with no interpretable cardiovascular context, duplicated a more complete publication, or provided insufficient methodological information for meaningful interpretation. Titles and abstracts were screened for relevance, followed by full‐text assessment and qualitative synthesis. Because this is a narrative review, no PRISMA flow count or formal pooled risk‐of‐bias estimate was generated. Evidence was interpreted hierarchically: human interventional and longitudinal outcome data were given the greatest weight for clinical inference; cross‐sectional human studies were treated as associative; and animal or cell experiments were used to support mechanistic plausibility. Causal language was reserved for experimental manipulations that directly tested a pathway and was not extrapolated to clinical causality without corresponding human evidence.

## 3 Cellular Mechanisms Linking Gut Microbiota Diversity to CVDs Progression

### 3.1 Microbiota‐Immune System Interactions

The gut microbiota exerts a profound influence on the host immune system, shaping both local and systemic immune responses. This bidirectional communication is critical for maintaining immune homeostasis, but dysbiosis can trigger chronic inflammation, a known driver of atherosclerosis and other CVDs [19]. Microbes and their metabolites interact with immune cells, affecting their differentiation, activation, and cytokine production. The GALT represents the largest lymphoid organ in the body, continuously interacting with commensal bacteria and dietary antigens [20]. This constant exposure trains the immune system, promoting tolerance to beneficial microbes while mounting responses against pathogens. Changes in gut microbiota composition, or dysbiosis, can compromise the integrity of the intestinal barrier, leading to increased permeability, often termed “leaky gut” [21].

When the gut barrier is compromised, bacterial components such as LPS and other pathogen‐associated molecular patterns (PAMPs) can translocate into the systemic circulation. This systemic exposure triggers an inflammatory response by activating Toll‐like receptors (TLRs) on various immune cells, including macrophages and endothelial cells [22]. The resulting chronic low‐grade inflammation, characterized by elevated levels of pro‐inflammatory cytokines like IL‐6 and C‐reactive protein (CRP), fuels endothelial dysfunction and accelerates atherosclerotic plaque formation and progression. Studies indicate an inverse relationship between gut microbial diversity and inflammatory markers such as high‐sensitivity CRP (hsCRP) and IL‐6 [23, 24].

Regulatory T cells (Tregs) are a subset of CD4^+^ T lymphocytes that are critical for maintaining immune tolerance and suppressing excessive immune responses. The gut microbiota significantly influences Treg development and function, particularly in the intestinal lamina propria. Specific microbial species and their metabolites, such as SCFAs, can promote the differentiation and expansion of Tregs [25]. For instance, Bifidobacterium, a commonly used probiotic, has been shown to alter the gut microbiota systematically in a Treg‐dependent manner in murine models. This alteration enhances the mitochondrial fitness and IL‐10‐mediated suppressive functions of intestinal Tregs, which can mitigate inflammatory conditions [26]. Conversely, dysbiosis leading to a reduction in Treg‐inducing bacteria can impair immune regulation, contributing to chronic inflammation and autoimmunity, which are comorbidities associated with accelerated atherosclerosis. The balance between pro‐inflammatory Th17 cells and anti‐inflammatory Tregs is a key determinant of the inflammatory state, and gut microbiota composition directly affects this balance (Table 1), thereby influencing cardiovascular health [27, 28].

**Table 1:** Microbiota‐immune interactions and potential relevance to atherosclerosis, ACS, and PAD.

Axis of interaction	Key mechanisms and molecular players	Impact on vascular pathophysiology	Potential links to clinical syndromes
Microbiota‐immune system crosstalk in the gut lumen and epithelium	MAMP recognition: TLRs (e.g., TLR2/4, TLR5) sensing bacterial LPS, flagellin. Metabolite signaling: SCFAs (butyrate, propionate) via GPCRs (GPR41, GPR43) and HDAC inhibition. Bile acid metabolism: Secondary BAs (e.g., LCA, DCA) as signaling molecules via FXR and TGR5. • Intestinal barrier integrity: Regulation of mucin (MUC2) production and tight junction proteins (occludin, ZO‐1).	Systemic endotoxemia: Translocation of microbial products (e.g., LPS) into circulation, triggering innate immune activation. Priming of myeloid cells: Enhanced monocyte/neutrophil reactivity and inflammasome activation (NLRP3). Metabolic inflammation: Insulin resistance and adipose tissue inflammation.	Atherosclerosis initiation and progression:
			Endothelial dysfunction. Increased oxidative stress. Foam cell formation [20, 21, 23, 25, 26].
GALT and systemic immune tone	Dendritic cell education: Lamina propria DCs sample microbiota, shaping T cell polarization. IgA class‐switch recombination: Microbiota‐dependent IgA coating of commensals, preventing translocation. Generation of circulatory immune intermediates: Gut‐primed pro‐inflammatory Th1/Th17 cells and B2 cells migrating to distant sites.	Chronic, low‐grade systemic inflammation: Elevated IL‐6, TNF‐α, and IL‐17. Plaque instability: Increased infiltration of inflammatory leukocytes into the arterial intima and adventitia. Hematopoietic stem/progenitor cell (HSPC) activation: Myelopoiesis in bone marrow and splenic extramedullary hematopoiesis.	ACS:
			Promotion of plaque rupture/erosion. Pro‐thrombotic state (tissue factor expression, platelet hyperreactivity) [20, 21, 22, 23, 25, 26].
Tregs and loss of immune homeostasis	Induction and function of colonic Tregs: Microbiota‐derived antigens and SCFAs (esp. butyrate) promote Foxp3+ Treg differentiation and function via epigenetic modification (HDAC9 inhibition). Peripheral Treg generation: Aryl hydrocarbon receptor (AhR) ligands from tryptophan metabolism. Treg/Th17 balance: Disruption by dysbiotic taxa (e.g., reduction in *Faecalibacterium prausnitzii*).	Failure of inflammatory resolution: Unchecked effector T cell and macrophage activity in plaques. Vascular Treg deficiency: Impaired suppression of endothelial activation and smooth muscle cell proliferation. Systemic autoimmunity: Generation of auto‐antibodies (e.g., anti‐HSP60, anti‐oxLDL).	Atherosclerosis Progression and PAD: Accelerated plaque growth. Impaired collateralization (angiogenesis). Increased risk of restenosis and critical limb ischemia [17, 20, 21, 22, 23, 25, 26].
Integrative clinical translation and therapeutic implications	Biomarker potential: Plasma TMAO, LPS‐binding protein (LBP), SCFA profiles, microbiota diversity indices (Shannon, Faith's PD). Intervention targets:	Modulation of disease trajectory:	Across the spectrum:
	Pre/pro/synbiotics: Selective fiber, *Akkermansia muciniphila*, and so forth. Pharmacologic: FXR/TGR5 agonists, HDAC inhibitors, tryptophan derivatives. Fecal microbiota transplantation (FMT): For the modulation of the ecosystem.	Stabilization of atherosclerotic plaques. Reduction in systemic inflammatory burden. Improvement in endothelial function and vascular repair mechanisms.	Primary prevention of atherosclerosis. Secondary prevention post‐ACS. Slowing PAD progression and reducing amputation risk [20, 21, 22, 23, 25, 26, 27].

### 3.2 Endothelial Dysfunction and Vascular Remodeling

Endothelial dysfunction represents an early, pivotal event in the development of atherosclerosis. It is characterized by impaired nitric oxide (NO) bioavailability, increased oxidative stress, adhesion molecule expression, and enhanced vascular permeability. The gut microbiota and its metabolites directly or indirectly affect endothelial health, thereby influencing vascular remodeling and disease progression [29, 30].

**Figure 1:**
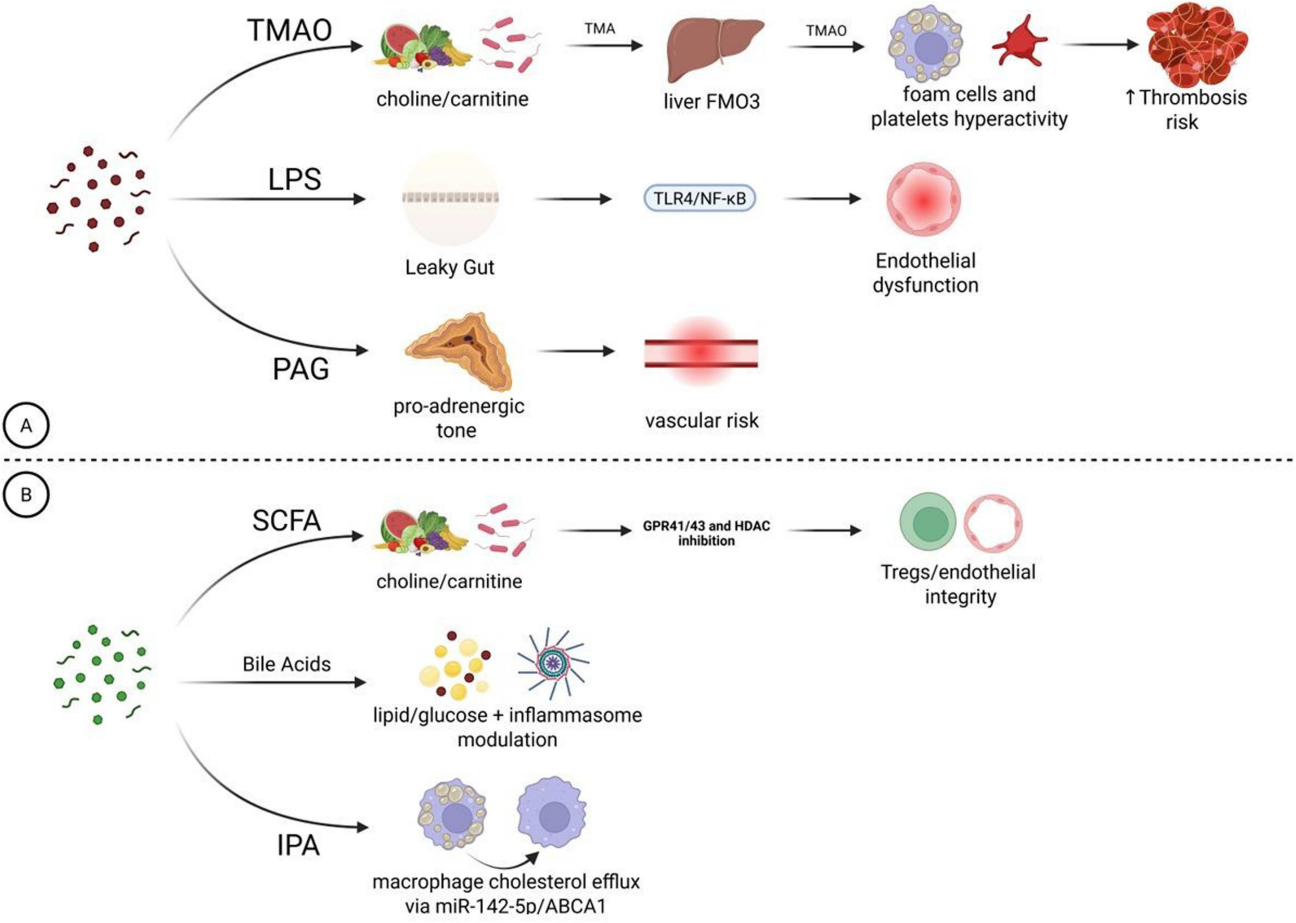
Microbiota‐derived mediators linked to atherothrombotic risk. (A) Dysbiosis‐associated pathways include TMA/TMAO generation, LPS translocation, inflammatory signaling, foam‐cell formation, and platelet reactivity. (B) A higher‐diversity, SCFA‐producing state is associated with barrier integrity, immunoregulation, and potentially protective bile‐acid and tryptophan‐metabolite signaling. The diagram summarizes mechanistic evidence; several links remain preclinical.

Numerous microbial metabolites are linked to endothelial function (Figure 1). TMAO, generated after gut microbial metabolism of trimethylamine (TMA)‐containing precursors such as l‐carnitine and phosphatidylcholine followed by hepatic oxidation, has been associated with atherothrombotic risk. Experimental studies indicate that TMAO can promote oxidative stress and inflammatory signaling and can impair NO bioavailability, whereas human studies predominantly show associations between higher TMAO levels and adverse cardiovascular outcomes [31]. Conversely, SCFAs, particularly butyrate, acetate, and propionate, are generally considered potentially protective metabolites (Table 2). Experimental and physiological studies suggest that SCFAs can support endothelial barrier integrity, modulate blood pressure and inflammation, and influence metabolic homeostasis [32]. Butyrate also acts as a histone deacetylase (HDAC) inhibitor and energy source for colonocytes, which may indirectly reduce systemic inflammatory exposure. Phenylacetylglutamine (PAGln), another gut microbiota‐derived metabolite, has been associated with future coronary risk and has demonstrated adrenergic receptor‐mediated effects in experimental models [33, 34]. These findings support mechanistic plausibility but do not imply that metabolite concentrations alone establish clinical causality.

**Table 2:** Microbiota‐derived metabolites and vascular remodeling pathways relevant to atherosclerotic disease.

Pathophysiological axis and key microbial metabolites	Molecular mechanisms and signaling pathways in the vasculature	Impact on vascular structure and function	Contribution to clinical syndromes
Axis of endothelial dysfunction and activation	TMAO: Inhibits endothelial nitric oxide synthase (eNOS) via S6K1‐mediated phosphorylation; induces NLRP3 inflammasome activation in ECs. Impaired SCFA signaling (butyrate/propionate): Loss of HDAC inhibition leads to upregulation of NF‐κB and AP‐1, promoting a pro‐adhesive endothelial phenotype. Secondary bile acids (e.g., LCA): At pathological concentrations, induce endothelial oxidative stress via ROS generation and mitochondrial dysfunction.	Reduced NO bioavailability: Impaired vasodilation, increased vascular tone. Pro‐inflammatory endothelium: Upregulation of VCAM‐1, ICAM‐1, E‐selectin. Pro‐thrombotic shift: Increased tissue factor (TF) and von Willebrand factor (vWF) expression; reduced thrombomodulin.	Atherosclerosis initiation: Primary site for leukocyte adhesion and subendothelial lipid accumulation. ACS Trigger: Endothelial erosion as a precipitant of acute thrombosis. PAD: Contributes to ischemic symptoms and impaired tissue perfusion [29, 31, 32, 33, 34].
Axis of vascular smooth muscle cell (VSMC) phenotype switching and remodeling	TMAO and Phenylacetylglutamine: Activate RhoA/ROCK and α‐adrenergic receptor pathways, promoting VSMC contraction, migration, and synthetic phenotype shift. SCFA Deficiency: Loss of suppression on HDAC6/9, leading to increased KLF5 expression and VSMC proliferation. Imidazole propionate: Impairs insulin/Akt signaling in VSMCs, promoting a pro‐inflammatory, proliferative state.	Neointimal hyperplasia: VSMC migration from media to intima, excessive proliferation, and ECM deposition. Plaque destabilization: Reduced fibrous cap formation due to impaired VSMC‐mediated collagen synthesis. Medial calcification and stiffness: VSMC osteogenic transition in PAD and CKD contexts.	Atherosclerosis progression: Central to plaque growth and fibrous cap integrity. In‐stent restenosis and PAD progression: Primary driver of luminal narrowing post‐revascularization and in native vessels [29, 31, 32, 33, 34].
Axis of adventitial and perivascular remodeling	Primary bile acids (in dysbiosis): Excessive accumulation can activate mast cells and adventitial fibroblasts via MRGPRX2/TLR4. SCFA Deficiency: Reduced GPR109A signaling on perivascular adipose tissue (PVAT) macrophages, fostering a pro‐inflammatory, adipokine‐dysregulated milieu.	Adventitial inflammation: Immune cell infiltration (macrophages, T cells, mast cells) driving outward remodeling and vasa vasorum neovascularization. Loss of PVAT anti‐contractile function: Contributes to increased vasoconstriction and vascular stiffness. Vasa vasorum dysregulation: Facilitates intraplaque hemorrhage and leukocyte trafficking.	Plaque vulnerability (ACS): Hypoxic, inflamed adventitia promotes plaque instability. PAD and arterial stiffness: Critical in medial calcification and compliance loss in distal arteries [29, 31, 32, 33, 34].
Axis of systemic metabolic and hormonal integration	TMAO: Amplifies angiotensin II receptor signaling and impairs renal handling of salt, potentiating hypertension. SCFA (Propionate): Mediates enteric gluconeogenesis signaling via gut‐brain neural circuits, influencing sympathetic tone. Indoxyl sulfate (uremic toxin): A tryptophan metabolite that induces endothelial senescence and VSMC osteoblast differentiation via p53 and NF‐κB.	Sustained hypertension: Chronic pressure overload accelerates endothelial injury and hypertrophic remodeling. Insulin resistance and hyperglycemia: Metabolic stress exacerbating endothelial dysfunction and oxidative stress. Accelerated vascular aging: Promotion of cellular senescence and vascular calcification.	Systemic accelerant: Potentiates all stages of atherosclerosis, ACS, and PAD across all vascular beds. Renal‐cardiovascular nexus: Links CKD (a major PAD risk factor) to accelerated vascular disease via gut‐derived uremic toxins [29, 31, 32, 33, 34].

The cellular signaling pathways involved in atherosclerosis are extensively influenced by gut microbiota‐derived factors. Endothelial cells, vascular smooth muscle cells, and macrophages are key cellular players whose functions are modulated [35]. For instance, TMAO activates the nuclear factor kappa‐light‐chain‐enhancer of activated B cells (NF‐κB) pathway in endothelial cells, leading to increased expression of adhesion molecules such as vascular cell adhesion molecule‐1 (VCAM‐1) and intercellular adhesion molecule‐1 (ICAM‐1), which facilitate the recruitment of monocytes to the arterial wall. This monocyte infiltration is a critical step in atherosclerotic plaque development [36, 37]. Moreover, TMAO also affects cholesterol metabolism within macrophages, promoting cholesterol accumulation and foam cell formation, another hallmark of early atherosclerosis. Indole‐3‐propionic acid (IPA), a solely microbially derived tryptophan metabolite, is downregulated in coronary artery disease patients and alleviates atherosclerotic plaque development in mouse models [38]. IPA promotes cholesterol efflux from macrophages via an miR‐142‐5p/ABCA1 signaling pathway, indicating a protective role for this metabolite. SCFAs, through activation of G‐protein coupled receptors (GPCRs) like GPR41 and GPR43, can modulate immune cell function and vascular tone, contributing to vascular protection. The intricate crosstalk between microbial metabolites and host cellular signaling pathways significantly contributes to the progression or protection against CVDs [39, 40].

### 3.3 Evidence Heterogeneity, Conflicting Findings, and Reverse Causality

Human microbiome studies do not identify a single reproducible CVD signature. Some cohorts and meta‐analyses report lower microbial richness or diversity in coronary disease, whereas other studies find little or no difference in overall alpha‐diversity but detect shifts in particular taxa, functional genes, or metabolites [5, 32]. Differences in population structure, stool collection, sequencing platform, bioinformatic pipeline, dietary exposure, geography, disease severity, and medication use can therefore generate apparently conflicting microbial profiles even when downstream functional pathways overlap.

Reverse causality is an additional concern. ACS, advanced PAD, hospitalization, ischemia, altered food intake, renal dysfunction, and treatment changes can themselves modify intestinal physiology and the gut microbiome. Similarly, circulating TMAO reflects not only microbial TMA production but also precursor intake, hepatic FMO3 activity, and renal clearance. Consequently, an association between a taxon or metabolite and CVD cannot be assumed to indicate that the microbial feature initiated the disease process [41, 42, 43, 44].

Accordingly, the evidence in this review is interpreted as a gradient rather than a binary causal/non‐causal classification. Reproducible prospective associations strengthen temporality; Mendelian‐randomization analyses can provide supportive genetic triangulation when their assumptions are satisfied; experimental models can test pathway necessity or sufficiency; and randomized human interventions provide the most direct evidence that modifying a microbial pathway changes a clinically relevant endpoint. For several epigenetic, plaque‐stability, and PAD‐specific mechanisms, human causal evidence remains limited despite strong biological plausibility [41, 42, 43].

## 4 Molecular Mechanisms Mediating Gut Microbiota Influence on Atherosclerosis, ACS, and PAD

### 4.1 Pro‐Atherogenic Metabolites: TMAO and Beyond

TMAO has received substantial attention for its consistent association with increased cardiovascular risk. TMAO is generated through a two‐step process involving specific gut microbial enzymes and a host enzyme. First, certain gut bacteria metabolize dietary compounds rich in TMA moieties, such as choline, l‐carnitine, and phosphatidylcholine, which are abundant in red meat, eggs, and dairy products. This microbial metabolism produces TMA [45, 46]. Subsequently, TMA is absorbed into the bloodstream and transported to the liver, where it is oxidized by flavin‐containing monooxygenases (primarily FMO3) into TMAO. The specific microbial taxa responsible for TMA production include species from genera like *Firmicutes* and *Proteobacteria* [47]. The diversity and activity of these TMA‐producing bacteria directly influence circulating TMAO levels. For instance, a diet rich in precursors like l‐carnitine can alter gut microbiota composition, promoting the growth of bacteria capable of converting l‐carnitine to TMA, thereby increasing TMAO production [48].

Elevated circulating TMAO levels have been associated with atherosclerosis and adverse cardiovascular outcomes, and experimental studies provide several plausible mechanisms (Table 3). In cell and animal models, TMAO can impair NO‐related endothelial function, increase oxidative and inflammatory signaling, alter cholesterol handling in macrophages, and enhance platelet responsiveness [49, 50, 51, 52]. These experimental findings are compatible with the epidemiological association between higher TMAO concentrations and major adverse cardiovascular events. However, most human outcome data remain observational, TMAO is influenced by renal function and diet, and it has not yet been established that selective lowering of TMAO itself reduces cardiovascular events. TMAO should therefore be interpreted as a mechanistically informative biomarker and potential therapeutic target rather than a proven independent causal driver in humans [41, 42, 43].

**Table 3:** TMAO‐related gut‐vascular pathways, disease associations, evidence gaps, and translational targets.

Component	Key mechanisms and pathways	Link to atherosclerosis	Link to ACS	Link to PAD progression	Innovative research/therapeutic targets
Precursor generation (gut microbiota)	Microbial diversity metrics: Decreased α‐diversity and altered β‐diversity correlate with enriched *CutC/D* (TMA‐lyase) gene clusters. Key taxa: *Prevotella*, *Bacteroides*, *Clostridia*, and *Enterobacteriaceae* shifts. Dietary choline/carnitine/PC conversion to TMA.	Shapes chronic endothelial inflammatory milieu.	May influence plaque vulnerability and trigger events via acute inflammatory bursts.	May contribute to systemic endothelial dysfunction affecting the coronary and peripheral vascular beds.	Pharmacomicrobiomics: Precision pre/probiotics, non‐absorbable TMA‐lyase inhibitors (e.g., iodomethylcholine), FMT targeting specific consortia [45, 46, 47].
Hepatic conversion (FMO3)	Host genetics: *FMO3* polymorphisms affect TMA → TMAOX conversion efficiency. Hormonal regulation: Bile acids and thyroid hormone modulate FMO3 expression.	Determines systemic TMAO load, a continuous risk variable.	Acute‐phase reactants may modulate FMO3, potentially elevating peri‐ACS TMAO.	Chronic elevation contributes to disease severity and CLI risk.	Genetic and Epigenetic Editing: CRISPR‐based *FMO3* modulation, siRNA targeting hepatic *FMO3* [45, 46, 47, 49].
Direct and indirect pro‐atherogenic mechanisms	Cellular: Scavenger receptor (CD36, SR‐A1) upregulation → foam cell formation. Signaling: MAPK/NF‐κB activation → vascular inflammation. Thrombotic: Enhanced platelet hyperreactivity via Ca^2+^ release. Metabolic: Bile acid disruption → reverse cholesterol transport impairment.	Potential contributor to intimal lipid accumulation and plaque initiation/growth.	Plaque rupture: Promotes thin‐cap fibroatheroma. Thrombosis: Amplifies platelet aggregation at rupture site.	Promotes atherosclerotic burden in femoro‐popliteal arteries. Impaired collateralization (angiogenesis).	Multi‐omics integration: Combining metabolomics (TMAO), proteomics (platelet receptors), and single‐cell transcriptomics of plaques [45, 46, 47, 49].
Systemic and vascular effects	Endothelial dysfunction: Reduced NO bioavailability, increased adhesion molecules (VCAM‐1/ICAM‐1). Vascular remodeling: Smooth muscle cell phenotypic switching, fibrosis. Renal crosstalk: Impaired renal excretion creates a positive feedback loop.	Chronic endothelial injury and vascular stiffening.	Acute endothelial activation facilitates leukocyte recruitment to unstable plaque.	Mediates ischemia‐reperfusion injury and endothelial dysfunction in microcirculation.	Advanced imaging biomarkers: PET‐MRI with TMAO‐tagged radiotracers for plaque activity. Renal clearance enhancers [45, 46, 47, 49, 50].
Clinical and translational correlates	Biomarker utility: Higher fasting plasma TMAO has been associated with MACE and adverse limb events in observational studies. Dietary modulation: Vegan/Mediterranean diets reduce TMAO; red meat/dairy increase. Comorbidity interaction: Synergy with CKD, diabetes, and heart failure.	Strong epidemiological association with coronary artery calcium score and plaque burden.	Elevated admission TMAO is associated with higher mortality, stent thrombosis, and recurrent MI.	Association with Rutherford class, amputation risk, and impaired walking performance.	Digital health integration: AI‐powered platforms using microbiome + TMAO + clinical data for dynamic risk stratification. Personalized nutrition apps with real‐time biomarker feedback [45, 46, 47, 51].
Unresolved questions and future directions	Chicken‐or‐egg: Does disease state alter microbiome, or vice versa? Circadian rhythm: Diurnal variation of TMAO production/effect? Microbiome resilience: Can a “healthy” microbiome resist TMAO elevation on a high‐risk diet?	Mechanistic studies in human‐derived organ‐on‐chip models with patient microbiome.	Role in plaque erosion versus rupture? Interaction with systemic stress (catecholamines).	Specificity for PAD versus CAD; role in vascular calcification patterns.	Synthetic biology: Engineered bacterial strains that degrade TMA. Bacteriophage therapy: Targeting specific TMA‐producing bacteria [45, 46, 47, 51, 52].

### 4.2 SCFAs, Bile Acids, and Vascular Protection

In contrast to the adverse associations reported for TMAO, SCFAs, and microbiota‐modified bile acids have shown potentially protective cardiovascular actions, particularly in experimental and mechanistic studies. SCFAs are end products of dietary fiber fermentation and are rapidly absorbed, with butyrate serving as a major energy source for colonocytes [53]. Butyrate can strengthen epithelial tight‐junction function and may thereby reduce translocation of inflammatory microbial products such as LPS. SCFAs can also signal through GPCRs such as GPR41 and GPR43 and inhibit HDACs, influencing immune‐cell differentiation, cytokine production, vascular tone, and metabolic regulation [54, 55, 56, 57]. These mechanisms are biologically plausible and supported by preclinical work, but the magnitude and clinical importance of direct SCFA‐mediated vascular protection in humans remain incompletely defined.

Bile acids, synthesized in the liver from cholesterol, undergo extensive metabolism by gut bacteria, forming a diverse pool of secondary bile acids. This microbial transformation significantly alters their signaling properties [58]. Bile acids act as signaling molecules, primarily through nuclear receptors such as farnesoid X receptor (FXR) and G‐protein‐coupled bile acid receptor 1 (TGR5). Activation of FXR in the liver and intestine regulates lipid and glucose metabolism. For example, FXR activation reduces hepatic triglyceride synthesis and increases reverse cholesterol transport [59]. Microbial modifications of bile acids, such as deconjugation and dihydroxylation, can modulate the potency and specificity of these receptor activations. Changes in gut microbiota composition can therefore alter the bile acid pool, influencing host lipid metabolism and systemic inflammation [60]. Dysbiosis‐induced alterations in bile acid profiles are implicated in dyslipidemia and metabolic syndrome, both significant risk factors for atherosclerosis (Table 4). Specific bacterial taxa, such as those within the *Clostridiales* order, are critical for the conversion of primary to secondary bile acids, underscoring the direct molecular connection between microbial diversity and host lipid regulation [61, 62].

**Table 4:** SCFAs, bile acids, microbial diversity, and dysbiosis in vascular protection or dysfunction.

Metabolite class	Key microbial producers/transformers	Primary mechanisms of action (molecular/cellular)	Integrated vascular and systemic effects (tissue/physiological)	Link to disease progression
SCFAs (e.g., Acetate, propionate, butyrate)	*Faecalibacterium prausnitzii*, *Roseburia* spp., *Eubacterium* spp., *Clostridium* clusters IV, XIVa	1. Epigenetic regulation: HDAC inhibition → upregulation of anti‐inflammatory genes (e.g., *IL‐10*, *FOXP3*). 2. Receptor‐mediated signaling: Agonists of GPCRs (GPR41, GPR43, Olfr78) → modulate leukocyte adhesion, endothelial function, and renin secretion. 3. Immunomodulation: Promotion of Treg differentiation; inhibition of NLRP3 inflammasome activation in macrophages. 4. Endothelial protection: Enhancement of endothelial NO synthase (eNOS) activity and barrier integrity.	Enhanced endothelial Function: Improved vasodilation, reduced endothelial permeability. Systemic anti‐inflammation: Reduced circulating IL‐1β, IL‐6, TNF‐α; attenuated leukocyte recruitment to plaque. Metabolic homeostasis: Improved insulin sensitivity, reduced hepatic lipogenesis. Plaque stabilization: Increased fibrous cap thickness via smooth muscle cell modulation.	AS: Attenuates initiation and slow progression via anti‐inflammatory and cholesterol‐static effects. ACS: Potential to reduce plaque vulnerability and rupture risk (stabilization). PAD: May improve endothelial function and collateralization in chronic ischemia; modulate systemic metabolic risk [43, 53, 54, 55, 56, 58, 59].
Primary and secondary bile acids	*Bacteroides*, *Clostridium* spp., *Lactobacillus*, *Bifidobacterium*, *Hydroxysteroid dehydrogenases (HSDH)‐expressing bacteria*	1. Nuclear receptor signaling: FXR and TGR5 agonists (e.g., DCA, LCA) → regulate bile acids synthesis, glucose/lipid metabolism, and vascular tone. 2. Inflammasome modulation: TGR5 signaling → inhibition of NF‐κB and NLRP3 pathways in macrophages. 3. Gut barrier integrity: FXR activation strengthens tight junctions, reducing endotoxemia (LPS translocation). 4. Vascular cell effects: TGR5 activation in endothelium → eNOS activation; in macrophages → reduced foam cell formation.	Systemic metabolic control: Reduced hepatic gluconeogenesis, increased energy expenditure, improved lipid profiles. Reduced systemic inflammation: Lower LPS‐driven TLR4 activation; diminished cytokine storm. Plaque modulation: Dual role: Certain secondary bile acids may be protective (TGR5‐mediated), while hydrophobic bile acids may promote inflammation if dysregulated. Vascular tone regulation: TGR5‐induced vasodilation in peripheral vessels.	AS: Modulates metabolic drivers (dyslipidemia, diabetes) and vascular inflammation. The bile acid pool composition is critical. ACS: Dysbiosis‐driven bile acids dysregulation may exacerbate plaque inflammation and instability. PAD: Influences both macrovascular function and microvascular perfusion; potential link to claudication severity [43, 53, 54, 55, 56, 58, 59].
Microbial diversity and metabolite crosstalk	High alpha‐diversity consortia (e.g., high *Firmicutes/Bacteroidetes* functional redundancy)	Functional redundancy and resilience: Maintains stable SCFA/bile acids production despite perturbations. Reduced pathogen invasion: Competitive exclusion limits proteobacteria expansion and endotoxin production. Optimal metabolic handoff: Cross‐feeding networks optimize substrate conversion to protective metabolites (e.g., primary → secondary bile acids; fiber → SCFAs). Systemic signaling integration: Balanced SCFA:bile acids ratio optimizes FXR, GPCR, and immune signaling networks.	Preserved gut‐vascular axis: Robust barrier function, minimal metabolic endotoxemia, balanced immune priming. Homeostatic metabolic phenotype: Improved glycemic control, favorable lipidomics, reduced visceral adiposity. Vascular resilience: Attenuated age‐ and diet‐related endothelial dysfunction; tempered inflammatory response to vascular injury.	AS: Foundation for all protective mechanisms; low diversity has been associated with greater plaque burden in some cohorts. ACS: Diversity loss linked to heightened systemic inflammation and incident MI risk. PAD: Associates with disease severity, progression rate, and post‐revascularization outcomes [43, 53, 54, 55, 56, 58, 59].
Dysbiosis and pathogenic shift	Low diversity and expansion of *Proteobacteria*, *Enterobacteriaceae*, *Desulfovibrionaceae*	Metabolite depletion: Reduced SCFA synthesis, impaired bile acid metabolism. Endotoxemia and systemic inflammation: Increased LPS translocation → persistent TLR4 activation → cytokine release. Pro‐inflammatory metabolite production: Generation of TMAO and other deleterious compounds (not in primary scope). Oxidative stress: Depletion of antioxidant microbial products.	Endothelial Dysfunction: eNOS uncoupling, increased adhesion molecule expression (VCAM‐1, ICAM‐1). Plaque destabilization: Increased intra‐plaque hemorrhage, neovascularization, and metalloproteinase activity. Hypercoagulability: Platelet hyperreactivity, increased tissue factor expression. Insulin resistance and dyslipidemia: Exacerbation of core metabolic syndrome features.	AS: May contribute to initiation and progression through inflammatory and metabolic pathways. ACS: Potentiates triggers for plaque rupture and coronary thrombosis; linked to poorer post‐MI outcomes. PAD: Accelerates disease progression, increases amputation risk, and impairs wound healing [43, 53, 54, 55, 56, 58, 59].

## 5 Epigenetic Mechanisms Underpinning Microbiota–CVDs Associations

Emerging research indicates that the gut microbiota influences host physiology not only through direct metabolite signaling and immune modulation but also by inducing epigenetic modifications. These modifications, including DNA methylation, histone acetylation/methylation, and microRNA (miRNA) regulation, alter gene expression without changing the underlying DNA sequence, providing a dynamic layer of control over host responses to microbial signals [63].

### 5.1 DNA Methylation Changes Induced by Microbial Metabolites

DNA methylation, primarily occurring at CpG dinucleotides, is a fundamental epigenetic mechanism that regulates gene transcription. Microbial metabolites can directly or indirectly influence DNA methylation patterns in host cells [64]. SCFAs, particularly butyrate, can act as inhibitors of HDACs, enzymes that remove acetyl groups from histones. While primarily known for histone modification, HDAC inhibitors can also influence DNA methylation by altering chromatin accessibility and the activity of DNA methyltransferases (DNMTs) [65]. More directly, certain microbial metabolites affect the availability of substrates for methylation reactions. For example, folate and methionine, essential for the one‐carbon metabolism cycle that generates S‐adenosylmethionine (SAM), the primary methyl donor for DNA methylation, can be produced or modulated by gut bacteria [66, 67]. Alterations in gut microbiota composition can therefore impact SAM levels, potentially leading to widespread changes in DNA methylation patterns across the host genome. In the context of CVDs, aberrant DNA methylation in genes related to lipid metabolism, inflammation, and endothelial function can contribute to pathology [68, 69]. For instance, differential methylation of genes involved in cholesterol efflux or inflammatory cytokine production could promote atherosclerosis (Table 5).

**Table 5:** Proposed links between microbial metabolites, DNA methylation, and vascular disease phenotypes.

Microbial metabolite class and examples	Primary metabolic source(s)	Target cell/tissue in vasculature	Key epigenetic enzymes/complexes affected	Specific genomic targets/pathways epigenetically modified	Proposed mechanistic link to disease phenotype (atherosclerosis, ACS, PAD)
SCFAs (e.g., butyrate, propionate)	Dietary fiber fermentation by *Faecalibacterium*, *Roseburia*, and so forth.	Vascular endothelial cells, smooth muscle cells, monocytes/macrophages, T lymphocytes	Inhibition: HDACs (Class I/IIa). Activation: HATs (via HDACi effect). Substrate for: DNA methyltransferases?	*Hypomethylation/activation:* NFKBIA (IκBα), NOS3 (eNOS), anti‐inflammatory cytokine genes. *Hyper‐methylation/repression:* Pro‐inflammatory adhesion molecule genes (e.g., VCAM1).	Atherosclerosis: Enhanced endothelial integrity, reduced leukocyte adhesion. ACS/PAD: Plaque stabilization via increased fibrous cap strength; modulation of Treg/Th17 balance [64, 65, 66, 69].
TMAO (Precursor: TMA)	Choline/l‐carnitine metabolism by *Emergencia timonensis*, *Desulfovibrio*, and so forth.	Hepatocytes, macrophages, platelets, endothelial cells	Alteration of: DNMT activity (putative), SAM/SAH ratio perturbation.	*Hypermethylation/repression:* ABCA1, ABCG1 (cholesterol efflux); BMP2 (calcification inhibition). *Hypomethylation/activation:* Scavenger receptors (e.g., CD36, SR‐A1).	Atherosclerosis: Foam cell formation, impaired reverse cholesterol transport. ACS: Enhanced platelet hyperreactivity and thrombosis risk. PAD: Promotion of vascular calcification [64, 65, 66, 69].
Bile acid derivatives (e.g., Deoxycholic acid (DCA), Ursodeoxycholic acid (UDCA))	Primary bile acid metabolism by *Bacteroides*, *Clostridium*, and so forth.	Hepatocytes, vascular smooth muscle cells, immune cells	Modulation of: DNMT1/3a expression via FXR/TGR5 signaling.	*Hypermethylation/repression:* CYP7A1 (bile acid synthesis); SIRT1 (endothelial protection). *Hypomethylation/activation:* Pro‐fibrotic genes (TGFB1, COL1A1).	Atherosclerosis: Altered lipid metabolism, VSMC phenotypic switching to synthetic/pro‐fibrotic state. PAD: Contribution to medial fibrosis and arterial stiffness [64, 65, 66, 69].
Polyphenol metabolites (e.g., urolithins, equol)	Ellagitannins, isoflavones metabolism by *Gordonibacter*, *Adlercreutzia*, and so forth.	Endothelial cells, monocytes/macrophages	Activation: Ten‐eleven translocation (TET) dioxygenases; Inhibition: DNMTs.	*Hypomethylation/activation:* KLF2 (flow‐responsive transcription factor), NRF2 (antioxidant response). *Hypermethylation/repression:* TLR4 (innate immune signaling).	Atherosclerosis: Enhanced antioxidant and anti‐inflammatory endothelial phenotype. ACS/PAD: Improved endothelial function and reduced oxidative stress in ischemic tissues [64, 65, 66, 69].
Imidazole propionate (ImP)	Histidine metabolism by *Klebsiella* and *E. coli* in dysbiosis	Endothelial cells, adipocytes, hepatocytes	Impairment of: mTORC1 signaling, leading to altered DNMT expression.	*Hypermethylation/repression:* Insulin receptor substrate (IRS1) pathway genes.	Atherosclerosis/ACS/PAD: Exacerbation of insulin resistance as a key metabolic driver of endothelial dysfunction and plaque vulnerability [64, 65, 66, 69].
LPS/Endotoxin	Outer membrane of Gram‐negative bacteria (e.g., *Enterobacteriaceae*).	Monocytes/macrophages, endothelial cells	Priming of: Inflammatory gene loci via TLR4/NF‐κB‐driven changes in histone marks and DNMT recruitment.	*Hypomethylation/activation:* IL6, TNF, MMP9 (matrix degradation). *Bivalent domain resolution* at inflammatory promoters.	Atherosclerosis: Sustained pro‐inflammatory macrophage phenotype (M1), plaque inflammation. ACS: Plaque rupture via MMP activity. PAD: Contribution to systemic and local vascular inflammation [64, 65, 66, 69].

### 5.2 Histone Modification Pathways Influenced by Microbiota‐Derived Factors

Histone modifications, such as acetylation, methylation, phosphorylation, and ubiquitination, alter chromatin structure and regulate gene accessibility and expression. Gut microbiota‐derived metabolites are potent modulators of these processes. As mentioned, SCFAs, especially butyrate, are well‐known HDAC inhibitors [70]. By inhibiting HDACs, butyrate increases histone acetylation, leading to a more open chromatin structure and enhanced gene transcription. This mechanism is implicated in the anti‐inflammatory effects of butyrate, promoting the expression of anti‐inflammatory genes and suppressing pro‐inflammatory pathways in immune cells and endothelial cells [71]. For example, butyrate‐induced histone acetylation can suppress NF‐κB signaling, reducing the inflammatory burden on the vasculature. Beyond acetylation, microbial metabolites can also influence histone methylation [72]. The availability of methionine, again modulated by gut bacteria, affects the production of SAM, which is critical for histone methyltransferase activity. Thus, alterations in the gut microbiota's metabolic output can lead to shifts in histone methylation marks, affecting the expression of genes involved in vascular health and disease [73, 74]. These epigenetic changes can contribute to long‐term alterations in cellular function, predisposing individuals to or protecting them from CVDs (Table 6).

**Table 6:** Proposed histone‐modification pathways linking microbial metabolites to vascular and immune‐cell phenotypes.

Microbial metabolite class and examples	Proposed/identified histone modification target	Molecular mechanism and effector cells	Functional consequence in vascular pathobiology	Link to disease progression and microbial diversity
SCFAs (e.g., butyrate, propionate)	H3/H4 acetylation (H3K9ac, H3K27ac) HDAC inhibition (Class I/IIa)	Butyrate acts as an HDAC inhibitor in monocytes/macrophages, endothelial cells (ECs), and smooth muscle cells (SMCs). Increases chromatin accessibility of regulatory regions.	Atherogenesis: Polarizes macrophages to an anti‐inflammatory, athero‐protective phenotype (M2‐like). Enhances endothelial barrier function. Plaque stability: May promote fibrous cap stability via SMC phenotype modulation.	Diversity link: High‐fiber diet → diverse SCFA‐producing taxa (e.g., *Faecalibacterium*, *Roseburia*). Loss of diversity reduces SCFA pool, diminishing athero‐protective epigenetic signaling, potentially accelerating Atherosclerosis and increasing vulnerability in ACS [70, 71, 72, 73, 74].
TMAO (Derived from choline/carnitine)	H3K4me3 activation H3K9me3 repression	TMAO enhances pro‐inflammatory gene transcription in macrophages via H3K4 methyltransferase (e.g., SET7/9). May recruit repressive complexes at protective loci.	Plaque inflammation: Potentiates NLRP3 inflammasome activation and M1 macrophage polarization. Endothelial dysfunction: Suppresses protective gene expression (e.g., *NOS3*). Thrombosis: Promotes platelet hyperreactivity.	Diversity link: Enriched *Emergencia timonensis* and reduced *butyrate‐producers*. Creates a “pro‐atherogenic” epigenetic milieu. Strongly associated with ACS risk and PAD progression via enhanced inflammation and thrombosis [70, 71, 72, 73, 74].
Bile acid derivatives (e.g., secondary BAs: DCA, LCA; taurine‐conjugated)	H3K27ac modulation Regulation of H3K27me3 via PRC2	Activate nuclear receptors (FXR, VDR) in hepatocytes and immune cells, recruiting histone‐modifying co‐activators (e.g., p300) or repressors (EZH2).	Metabolic inflammation: Modulates hepatic and systemic inflammation. Influences cholesterol homeostasis via epigenetic regulation of *CYP7A1*. Vascular calcification: Specific BAs may alter osteogenic programs in vascular SMCs via H3K27me3.	Diversity link: BA metabolism requires specific bacterial enzymes (e.g., *bai* operon in *Clostridium* spp.). Dysbiosis alters the BA pool, shifting epigenetic regulation toward pro‐inflammatory and pro‐calcific states, relevant in advanced Atherosclerosis and PAD with calcification [70, 71, 72, 73, 74].
Polyamines (e.g., spermidine)	Hypusination of eIF5A (non‐canonical) Potential modulation of H3K4me3	Spermidine is a substrate for hypusine synthesis, crucial for the translation of specific mRNAs. Also serves as a lysine mimic, potentially inhibiting histone acetyltransferases (HATs).	Cellular senescence and autophagy: Promotes autophagy in ECs and SMCs, an athero‐protective process. May reduce senescence‐associated secretory phenotype (SASP).	Diversity link: Produced by various commensals (e.g., *Bifidobacterium*). Aging and low‐diversity microbiomes show reduced polyamine levels, contributing to vascular aging epigenetics, impacting all three diseases, particularly in PAD progression in the elderly [70, 71, 72, 73, 74].
Phenylacetylglutamine (PAGln) (and other phenylalanine derivatives)	H3K9ac/H3K27ac via adrenergic receptor signaling	Metabolite acts as a G‐protein coupled receptor ligand, triggering intracellular kinase cascades (PKA, PKC) that phosphorylate and activate histone‐modifying enzymes like p300/CBP in platelets and leukocytes.	Thrombosis and inflammation: Potentiates platelet activation and adhesion potential via epigenetic priming of pro‐thrombotic pathways. Enhances leukocyte adhesion to endothelium.	Diversity link: Generated by microbial metabolism of dietary phenylalanine. Dysbiosis with over‐representation of proteolytic bacteria increases PAGln, providing a novel epigenetic link between diet, gut flora, and thrombotic complications in ACS and ischemic PAD events [70, 71, 72, 73, 74].
Indole derivatives (e.g., indole‐3‐propionic acid, IPA)	AHR‐mediated histone modifications (e.g., H3K4me1 at enhancers)	Ligand for aryl hydrocarbon receptor (AHR). AHR translocates to the nucleus, forms a complex with transcriptional co‐regulators that possess histone‐modifying activity, shaping enhancer landscapes in ECs and T‐regs.	Antioxidant and anti‐inflammatory: Potentiates endothelial antioxidant defenses. Promotes anti‐inflammatory T‐regulatory cell differentiation. Strengthens gut barrier, reducing endotoxemia.	Diversity link: Derived from tryptophan by symbionts (e.g., *Clostridium sporogenes*). High microbial diversity supports IPA production, maintaining an anti‐inflammatory vascular epigenetic tone, protecting against inflammatory progression in Atherosclerosis [70, 71, 72, 73, 74].

### 5.3 MicroRNAs as Mediators in Cardiovascular Pathogenesis

miRNAs are small non‐coding RNAs that regulate gene expression post‐transcriptionally by targeting messenger RNAs (mRNAs) for degradation or translational repression. They are crucial regulators of various biological processes, including inflammation, lipid metabolism, and endothelial function, all central to CVDs [75, 76]. The gut microbiota can influence host miRNA expression, thereby mediating its effects on cardiovascular pathogenesis. For instance, specific microbial metabolites or bacterial components can induce changes in miRNA profiles within host cells, including those in the gut, liver, and vascular tissues [77]. These altered miRNA expressions can then impact the stability and translation of target mRNAs, leading to downstream physiological consequences. An example is the microbial metabolite IPA, which has been shown to alleviate atherosclerosis by promoting cholesterol efflux from macrophages through the regulation of miR‐142‐5p/ABCA1 signaling [78, 79]. Reduced IPA production contributes to aberrant overexpression of miR‐142‐5p, which in turn impairs ABCA1‐mediated cholesterol efflux, accelerating atherosclerosis [80]. Furthermore, immune cells, whose functions are heavily modulated by the gut microbiota, exhibit distinct miRNA signatures that influence their inflammatory potential [81]. Dysbiosis‐induced changes in immune cell activation and differentiation can therefore indirectly alter miRNA expression profiles that contribute to cardiovascular inflammation and plaque development [41]. The complex interplay between m6A RNA methylation regulators and miRNAs in coronary heart disease further underscores the epigenetic regulatory complexity (Table 7).

**Table 7:** Proposed metabolite‐miRNA pathways relevant to atherosclerosis, ACS, and PAD; many links remain mechanistic or exploratory.

Primary microbial metabolite class and examples	Source microbiota (example genera)	Target host cell/tissue	Induced/repressed microRNAs (key examples)	Mechanistic role of miRNA in disease pathway	Downstream gene targets and affected pathways	Clinical disease correlation and proposed biomarker potential
SCFAs	*Faecalibacterium*, *Roseburia*, *Clostridium* clusters IV, XIVa	Intestinal epithelium, immune cells (macrophages, Tregs), vascular endothelium	Induced: miR‐10a, miR‐181b Repressed: miR‐92a, miR‐33	Enhances endothelial stability and anti‐inflammatory signaling; promotes cholesterol efflux and M2 macrophage polarization.	ABCA1/G1, *KLF4*, *NF‐κB*, *SIRT1* → Cholesterol homeostasis, inflammation resolution	Atherosclerosis progression: Inverse correlation with plaque instability. Biomarker: Serum miR‐10a levels linked to butyrate production [41, 77, 78, 79, 80, 81].
TMAO	*Prevotella*, *Bacteroides*, *Desulfovibrio* (via hepatic oxidation)	Hepatocytes, platelets, vascular smooth muscle cells (VSMCs), endothelium	Induced: miR‐21‐5p, miR‐30c, miR‐223 Repressed: miR‐148a, miR‐26b	Promotes pro‐inflammatory macrophage phenotype, VSMC migration/proliferation, enhances platelet hyperreactivity.	*PPARα*, *ABCA1*, *SMAD7*, *CXCR4* → Foam cell formation, fibrosis, thrombosis	ACS: Strong predictor of major adverse cardiac events (MACE). Biomarker: Plasma miR‐30c correlates with TMAO and plaque rupture risk [41, 77, 78, 79, 80, 81].
Bile acid	*Clostridium scindens*, *Bacteroides*, *Lactobacillus*	Enterocytes, hepatocytes, immune cells in plaque	Induced: miR‐144, miR‐122 Repressed: miR‐33a, miR‐206	Modulates hepatic and systemic lipid metabolism, FXR/TGR5 signaling, and influences inflammasome activation.	ABCG5/8, *CYP7A1*, *FXR*, *NLRP3* → Bile acid homeostasis, lipoprotein metabolism, Inflammation	PAD progression: Linked to lipid profile dysregulation and endothelial dysfunction. Biomarker: Fecal BA profile and serum miR‐144 as combo markers [41, 77, 78, 79, 80, 81].
Polyamines (e.g., Spermidine, Putrescine)	*Bifidobacterium*, *Lactobacillus*, *Bacteroides*	Endothelial cells, cardiomyocytes, macrophages	Induced: miR‐221‐3p, let‐7 family Repressed: miR‐195, miR‐34a	Promotes autophagy, cellular longevity, and improves endothelial function; anti‐senescence effects.	*ATG5*, *p21*, *SIRT1*, BCL‐2 → Autophagy, senescence, oxidative stress response	AS and PAD: Associated with vascular aging and endothelial repair capacity. Biomarker: Low circulating spermidine and altered let‐7c in critical limb ischemia [41, 77, 78, 79, 80, 81].
LPS/Endotoxins (Gram‐negative bacteria)	*Enterobacteriaceae*, *Desulfovibrio*	Systemic circulation, monocytes/macrophages, endothelium	Induced: miR‐155, miR‐146a, miR‐34a Repressed: miR‐125b, miR‐181a	Drives potent pro‐inflammatory response via TLR4/NF‐κB; induces endothelial dysfunction and insulin resistance.	*SOCS1*, *IRAK1*, *TRAF6*, *SIRT1*, Bcl‐2 → Innate immune activation, endothelial apoptosis, metabolic endotoxemia	ACS and PAD progression: Links microbiota dysbiosis (“leaky gut”) to systemic inflammation and plaque vulnerability. Biomarker: LPS‐binding protein (LBP) with miR‐155 as inflammatory amplifier [41, 77, 78, 79, 80, 81].
Indole derivatives (e.g., Indole‐3‐propionic acid (IPA))	*Clostridium sporogenes*, *Bifidobacterium* spp.	Endothelium, aryl hydrocarbon receptor (AhR)+ immune cells	Induced: miR‐132, miR‐212 Repressed: miR‐142‐3p	Activates AhR for anti‐inflammatory signaling; strengthens endothelial barrier; antioxidant properties.	*PTEN*, PI3K/Akt, *Nrf2*, VCAM‐1 → Endothelial integrity, Oxidative stress reduction	AS stabilization, PAD: Potential protective role. Low IPA levels in metabolic syndrome. Biomarker: Urinary IPA metabolites and endothelial miR‐132 cluster [41, 77, 78, 79, 80, 81].

Interpretation of epigenetic evidence requires particular caution. Many proposed metabolite‐DNA methylation, histone, and miRNA relationships in Tables 5, 6, 7 are based on cellular systems, animal experiments, pathway inference, or studies outside direct human plaque tissue. These data are valuable for hypothesis generation, but longitudinal human validation and intervention studies showing that microbiome‐driven epigenetic changes alter cardiovascular outcomes are still limited.

## 6 Modifiable Risk Factors and Therapeutic Perspectives

**Figure 2:**
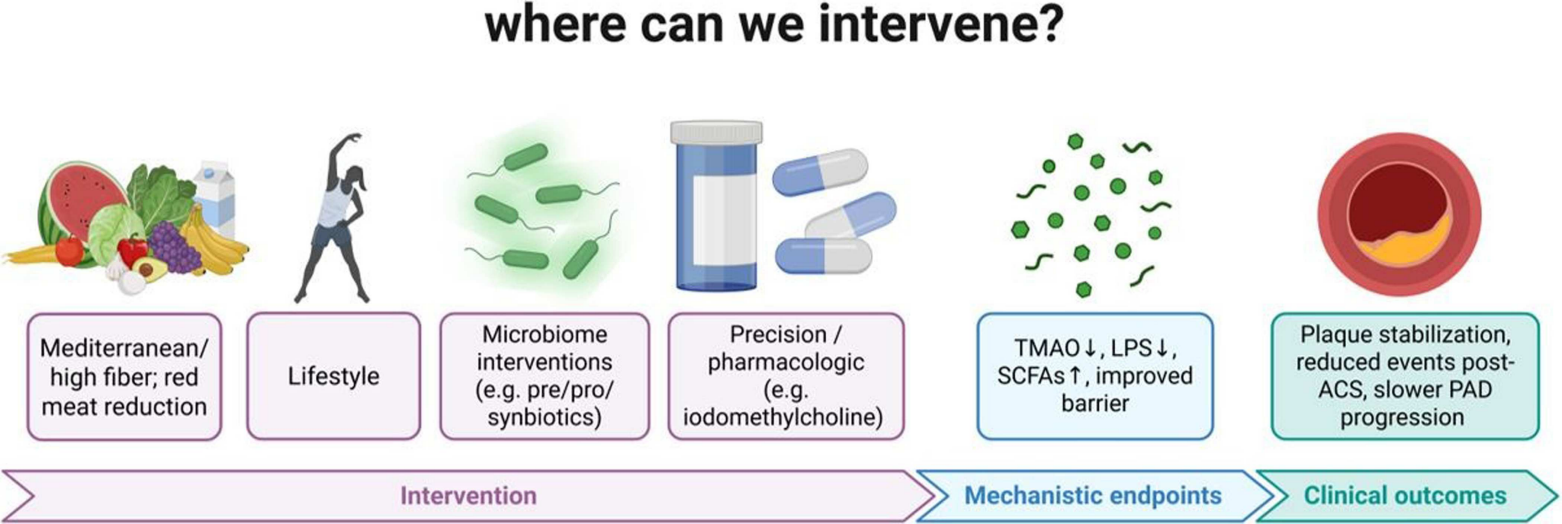
Investigational therapeutic entry points along the gut microbiota‐atherothrombosis axis. Dietary and lifestyle strategies, pre/pro/synbiotics, and targeted microbial‐metabolite interventions may alter TMAO, LPS, SCFAs, or barrier function. Effects on plaque stabilization, post‐ACS events, and PAD progression remain to be established in adequately powered clinical outcome trials.

Understanding the mechanistic links between gut microbiota and CVDs opens avenues for therapeutic interventions targeting the microbiome. Modifying risk factors and directly modulating microbial communities present promising strategies for prevention and management (Figure 2).

### 6.1 Diet, Lifestyle, and Microbiota Composition

Diet is a primary modulator of gut microbiota composition and function. Dietary patterns rich in fiber, fruits, vegetables, and whole grains (e.g., Mediterranean diet) promote a diverse microbiota dominated by beneficial, SCFA‐producing bacteria. These diets foster a microbial environment that supports gut barrier integrity, reduces systemic inflammation, and beneficially modulates lipid and glucose metabolism, thereby protecting against atherosclerosis [82, 83]. A personalized postprandial‐targeting (PPT) diet, for example, has been shown to induce more prominent changes to gut microbiome composition and alpha‐diversity compared to a Mediterranean diet, mediating associations between dietary changes and cardiometabolic outcomes like HbA1c, HDL‐C, and triglycerides [84]. Conversely, Westernized diets, high in saturated fats, red meat, and processed foods, can lead to dysbiosis, characterized by reduced diversity and an increase in pro‐inflammatory, TMA‐producing bacteria. A high enterolactone/low TMAO profile, linked to higher intake of whole grains and fiber and lower intake of red meats, is significantly associated with a lower risk of CAD [85]. Lifestyle factors beyond diet, such as physical activity, sleep, and stress management, also influence microbiota composition, further underscoring the holistic approach required for cardiovascular health. Weight management, through dietary and lifestyle changes, is a critical modulator, as body mass index is a prominent factor affecting gut microbiota composition [86].

#### 6.1.1 Demographic, Geographic, Dietary, and Medication‐Related Heterogeneity

Gut microbial composition is strongly context dependent, and this variability can obscure or exaggerate cardiovascular associations. Age, sex and hormonal state, host genetics or ancestry, body composition, diabetes, chronic kidney disease, smoking, alcohol exposure, physical activity, sleep, and socioeconomic or urbanization‐related factors can influence microbial community structure and metabolite production [32, 81, 85, 87]. Geographic and cultural differences are particularly important because habitual diet, food processing, fiber intake, animal‐protein exposure, sanitation, and environmental microbial exposures vary substantially between populations. A taxonomic signature identified in one country or dietary setting should therefore not be assumed to generalize across regions without external validation.

Concomitant medications are another major source of heterogeneity. Antibiotics can cause large short‐term perturbations, while commonly used cardiometabolic or gastrointestinal therapies, including statins, metformin, proton‐pump inhibitors, and other agents, may alter microbial composition or metabolite availability. Renal function is also crucial when interpreting metabolites such as TMAO because impaired clearance can raise circulating concentrations independently of microbial production. Future cardiovascular microbiome studies should prospectively record diet, geography, demographic variables, renal function, recent antibiotic or probiotic exposure, and concomitant medications, and should use stratified or multivariable analyses rather than attributing all observed differences directly to CVDs [42, 43, 87].

### 6.2 Probiotic, Prebiotic, and Synbiotic Interventions

Direct modulation of the gut microbiota using probiotics, prebiotics, and synbiotics presents a promising therapeutic strategy. Probiotics are live microorganisms that, when administered in adequate amounts, confer a health benefit on the host. Prebiotics are non‐digestible food ingredients that selectively stimulate the growth and/or activity of beneficial bacteria. Synbiotics combine both [88]. Clinical trials indicate that these interventions can reduce circulating levels of toxic metabolites associated with CVDs, such as endotoxin, indoxyl‐sulfate, and p‐cresyl sulfate. For instance, certain probiotic strains (e.g., *Lactobacillus* and *Bifidobacterium*) can reduce TMAO levels by competitively inhibiting TMA‐producing bacteria or by directly metabolizing TMA precursors [89, 90]. Prebiotic fibers, by promoting SCFA production, strengthen the gut barrier, reduce inflammation, and improve metabolic parameters. Supplementation with Bifidobacterium has been shown to alter the gut microbiota systematically, enhancing the suppressive functions of intestinal Tregs, which ameliorates colitis and suggests broader immune benefits relevant to cardiovascular health [91, 92]. However, the quality of many trials in this area has been noted as low, necessitating more robust, well‐designed studies (Table 8).

**Table 8:** Evidence and limitations of microbiome‐directed intervention classes relevant to atherosclerotic cardiovascular disease.

Intervention class	Representative approach	Current human evidence	Putative microbiome‐related mechanism	Key limitation/clinical status
Dietary pattern/fermentable fiber	Mediterranean‐ or plant‐forward patterns; whole grains; fermentable fiber	Human dietary studies support improvements in cardiometabolic risk factors; direct microbiome mediation of vascular outcomes remains difficult to isolate	Greater substrate availability for SCFA production; lower exposure to some TMA precursors; improved barrier‐related signaling	Diet is multi‐component; adherence and background diet are major confounders [81, 82, 88]
Probiotics	Selected *Lactobacillus* or *Bifidobacterium* strains	Small trials report strain‐specific changes in microbiota, inflammatory markers, lipids, or other surrogate endpoints	Potential effects on barrier integrity, immune signaling, metabolite competition, and SCFA‐related pathways	Marked strain/formulation heterogeneity; no established reduction in hard CVD outcomes [88, 89, 90]
Prebiotics	Inulin, resistant starch, and other fermentable substrates	Evidence is strongest for microbiome and cardiometabolic surrogate endpoints rather than cardiovascular events	Enrichment of fermentative taxa and increased SCFA production	Dose tolerance and large inter‐individual response; outcome evidence remains limited [88, 89, 90]
Synbiotics	Combined probiotic plus prebiotic formulations	Early and generally small human studies; post‐ACS studies are now entering the pipeline	Combined ecological support for selected strains and fermentative functions	No standardized formulation and insufficient cardiovascular outcome data [88, 89, 90]
Microbial enzyme/metabolite targeting	Inhibition of TMA‐producing pathways or downstream microbial‐metabolite signaling	Predominantly preclinical or early translational evidence	Selective reduction of pro‐atherogenic microbial products without broad microbiome depletion	Long‐term safety, target specificity, and event reduction are unproven [42, 43]
FMT, phage, engineered, or live biotherapeutics	Ecosystem transfer or targeted microbial engineering	Investigational for cardiovascular indications	Potential targeted remodeling of microbial community or function	Safety, durability, regulatory requirements, and clinical efficacy preclude routine CVD use [42, 43, 93]

### 6.3 Nutraceuticals and Pharmacological Modulation of Gut Microbiota‐Derived Pathways

Beyond direct microbial interventions, nutraceuticals and targeted pharmacological agents can modulate gut microbiota‐derived pathways. Nutraceuticals, such as polyphenols found in fruits, vegetables, and tea, are extensively metabolized by gut bacteria into bioactive compounds that possess antioxidant and anti‐inflammatory properties. These compounds can improve endothelial function and reduce atherosclerosis progression [94].

While broad‐spectrum antibiotics can disrupt the gut microbiota, targeted approaches may reduce specific pro‐atherogenic bacteria. However, the long‐term consequences of antibiotic use on gut health require careful consideration [84]. Lipid‐lowering medications like statins, commonly prescribed for CVD, have been shown to influence gut microbiota composition, suggesting an additional mechanism for their pleiotropic effects. The gut microbiota, in turn, may influence an individual's response to statin therapy [95]. Novel compounds designed to inhibit TMA lyases (bacterial enzymes responsible for TMA production) or hepatic FMO3 (the host enzyme converting TMA to TMAO) are under investigation as potential therapies to reduce TMAO levels and mitigate cardiovascular risk [96, 97]. Agents that alter bile acid signaling, such as FXR agonists, can influence lipid and glucose metabolism, offering another indirect route to cardiovascular protection through gut−liver interactions. These approaches highlight the potential for precision medicine strategies that consider the individual's gut microbiome profile when developing treatment plans for CVDs [98].

### 6.4 Clinical Implications for CVDs Prevention and Management

Circulating levels of microbiota‐derived metabolites, particularly TMAO, can serve as predictive biomarkers for cardiovascular event risk, potentially refining existing risk assessment models. A high enterolactone/low TMAO profile is associated with a lower CAD risk [99]. Emphasizing dietary patterns that promote beneficial gut microbiota (e.g., high‐fiber, plant‐based diets) represents a foundational strategy for CVD prevention. Clinicians can counsel patients on specific foods that influence microbiota composition and metabolite production, moving toward personalized nutrition [87]. Probiotics, prebiotics, and synbiotics, along with targeted pharmacological agents that inhibit TMA production or FMO3 activity, offer novel therapeutic avenues to modulate pro‐atherogenic pathways and reduce inflammation [100]. These interventions could complement traditional CVD treatments. Addressing gut dysbiosis could improve outcomes in conditions frequently comorbid with CVD, such as obesity, diabetes, and chronic kidney disease, where microbiota alterations contribute to systemic inflammation and metabolic dysfunction [81]. Integrating gut microbiome profiling into patient assessment could facilitate personalized therapeutic strategies, tailoring interventions to an individual's unique microbial signature and metabolic response. The integration of gut microbiota considerations into cardiovascular medicine presents a paradigm shift toward a more holistic and preventive approach to patient care [101, 102].

### 6.5 Ongoing Clinical Studies and the Translational Pipeline

Registered studies illustrate that cardiovascular microbiome research is moving from association toward interventional testing, but most current protocols remain pilot‐sized or emphasize mechanistic and surrogate outcomes rather than major cardiovascular events. Table 9 summarizes selected ongoing or planned studies with direct relevance to atherosclerosis, ACS, coronary disease, or host factors that modify cardiovascular microbiome relationships. Registry status was checked on ClinicalTrials.gov on August 11, 2026; the status and planned completion date may subsequently change.

**Table 9:** Selected ongoing or planned clinical studies relevant to microbiome‐based cardiovascular translation (ClinicalTrials.gov status checked August 11, 2026).

Registry/status	Population and design	Intervention or exposure	Microbiome/vascular endpoint	Translational question
NCT07411287 (SYMBIO‐ACS) Not yet recruiting	80 patients after recent ACS; randomized pilot trial	10‐week synbiotic (Pro‐B) plus guideline‐directed therapy versus guideline‐directed therapy	Primary: Change in plasma TMAO; inflammatory and cardiometabolic biomarkers	Can post‐ACS synbiotic modulation change a microbial metabolite target sufficiently to justify a larger prevention trial?
NCT07203846 (FLORA‐ACS) Not yet recruiting	50 post‐ACS patients on ticagrelor with high platelet reactivity; interventional study	7‐day rifaximin course	Platelet reactivity plus 16S rRNA‐based richness/diversity and follow‐up events	Does changing dysbiosis alter platelet reactivity, providing human mechanistic evidence for a gut‐platelet axis?
NCT06701968 (NORDHEART) Recruiting	150 patients with prior MI or chronic coronary syndrome; randomized parallel trial	Healthy nordic diet versus usual‐care dietary advice	Primary coronary plaque‐volume change at 18 months; gut microbiota and multi‐omics are exploratory endpoints	Are dietary effects on coronary plaque progression partly accompanied by microbiome changes?
NCT05135702 Not yet recruiting	20 patients with coronary artery disease; randomized crossover study	Sodium propionate versus placebo	Brachial‐artery flow‐mediated dilation; microbiome assessment is included	Can a microbiota‐related SCFA intervention acutely improve a human vascular surrogate endpoint?
NCT05334888 (XCVD) Recruiting	Prospective 2‐year cohort of 200 participants undergoing hormone therapy	Longitudinal sex‐hormone exposure	Changes in gut species abundance, metabolome/immunome, and cardiovascular risk markers	How do sex hormones and demographic context modify host‐microbiome cardiovascular risk relationships?
NCT06784557 Recruiting (registry last updated January 2025)	110 patients with atherosclerotic cardiovascular disease and dyslipidemia; randomized trial	Atorvastatin 40 mg versus atorvastatin 20 mg plus ezetimibe	Primary: change in gut microbiota; secondary lipids and inflammatory biomarkers	To what extent do common lipid‐lowering regimens themselves alter the gut microbiome and confound or mediate CVD associations?

## 7 Limitations, Future Perspectives, and Emerging Therapeutic Approaches

The current evidence base has several important limitations. Cardiovascular microbiome studies vary widely in geography, age, comorbidity, medication exposure, diet, sample handling, sequencing depth, taxonomic databases, and statistical pipelines. Cross‐sectional designs remain common, which limits temporal inference and increases the possibility of reverse causality. Stool samples may not represent mucosa‐associated or plaque‐associated microbial communities, and metabolite concentrations are influenced by host factors such as renal function and hepatic metabolism. Many proposed cellular and epigenetic mechanisms are supported mainly by preclinical studies, whereas human intervention trials are typically small and focus on surrogate endpoints. There is also no universally accepted definition of cardiovascular “dysbiosis,” making direct comparisons and clinical cutoffs premature [5, 10, 72, 93, 94].

### 7.1 Future Research Priorities

Progress toward causal inference will require large prospective cohorts with repeated microbiome and metabolite sampling before and after cardiovascular events, standardized pre‐analytical and bioinformatic methods, and detailed measurement of diet, geography, renal function, medications, and host genetics. Cross‐cohort replication and multi‐omics integration should be combined with causal triangulation approaches, including carefully interpreted Mendelian‐randomization analyses, mechanistic experiments, and randomized interventions. Trials should pre‐specify microbiome endpoints and, when feasible, measure plaque, limb, or cardiovascular event outcomes rather than relying solely on taxonomic shifts. PAD‐specific work is particularly needed because current evidence is much thinner than for coronary disease [103, 104, 105].

### 7.2 Emerging Therapeutic Approaches

Potential strategies include precision dietary and prebiotic interventions, strain‐defined probiotics or synbiotics, postbiotic or metabolite supplementation, selective inhibition of microbial TMA‐producing enzymes, modulation of bile‐acid or other microbial‐metabolite signaling, and more experimental approaches such as phage therapy, engineered microbial consortia, live biotherapeutic products, or fecal microbiota transplantation. A central translational advantage of pathway‐selective strategies is the possibility of modifying a harmful microbial function without broadly depleting the microbiome. Nevertheless, these approaches should be regarded as investigational in cardiovascular medicine. Before routine use, studies must establish durable target engagement, safety, reproducibility across populations, interaction with guideline‐directed cardiovascular medications, and, most importantly, improvement in clinically meaningful outcomes [42, 81, 92].

## 8 Conclusion

Current evidence supports a biologically plausible and clinically relevant association between gut microbial ecology, microbial metabolites, and the spectrum of atherosclerotic CVDs, including ACS and PAD. Experimental studies provide substantial mechanistic support for pathways involving barrier dysfunction, immune activation, endothelial signaling, platelet reactivity, TMAO, SCFAs, bile acids, and epigenetic regulation. However, the strength of clinical causal inference is uneven: many human studies remain observational, taxonomic signatures are heterogeneous, and host factors such as diet, geography, renal function, comorbidity, and medication use can modify both the microbiome and measured metabolites. Accordingly, microbiome features should not yet be treated as independent causal determinants or routine therapeutic targets solely on the basis of association. Dietary and microbiome‐directed interventions are promising, and ongoing trials are beginning to test whether modifying these pathways changes vascular or mechanistic endpoints, but evidence for the reduction of major cardiovascular events is not yet sufficient for routine microbiome‐based therapy. Future standardized, prospective, and adequately powered human studies that integrate longitudinal sampling, multi‐omics, causal triangulation, and clinically meaningful outcomes will be essential to determine which gut‐vascular pathways are actionable in personalized cardiovascular prevention and treatment.

## Author Contributions

Usamah Sayed, Zarina Nasirova, Quvonch Tursunov, Omer Qutaiba B. Allela, Lara Asim Kzar, Rajashree Panigrahi, and Neeraj Bainsal wrote the main manuscript text and prepared figures. Amr Ali Mohamed Abdelgawwad El‐Sehrawy reviewed the manuscript. All authors review the final version of the manuscript and confirm it. This paper is submitted with the agreement of all its co‐authors, and the author list has been approved by all authors.

## Funding

The authors have nothing to report.

## Conflicts of Interest

The authors declare no conflicts of interest.

## Data Availability

Data sharing is not applicable to this article as no data sets were generated or analyzed during the current study.
